# Pyoderma Gangrenosum Presentation After Abdominoplasty and Augmentation Mastopexy

**DOI:** 10.7759/cureus.58060

**Published:** 2024-04-11

**Authors:** Michele Champigny, Ariel Toomey, Daniel Sherbert

**Affiliations:** 1 Plastic and Reconstructive Surgery, Corewell Health William Beaumont University Hospital, Royal Oak, USA

**Keywords:** postoperative pyoderma gangrenosum, pyoderma gangrenosum post surgery, pyoderma gangrenosum of the breast, plastic and reconstructive surgery, pyoderma gangrenosum (pg)

## Abstract

Pyoderma gangrenosum is a rare ulcerative skin disease of uncertain etiology, which in some cases can be misdiagnosed as an infectious process. In even more unique cases, this can occur in the postoperative period. Termed postsurgical pyoderma gangrenosum, this type of inflammatory skin condition requires a high index of suspicion to be able to appropriately treat and reduce complications. We present a 55-year-old female who presented with multiple wounds following mastopexy and abdominoplasty. With a prompt diagnosis and a multidisciplinary approach, we could accurately care for the patient and minimize poor aesthetic sequela.

## Introduction

Pyoderma gangrenosum is a rare inflammatory, noninfectious, ulcerative neutrophilic skin disease of uncertain etiology, commonly misdiagnosed as an aggressive skin infection. According to a study by Xu et al., it is thought to have a prevalence of about 5.8 cases per 100,000 people, and the adjusted prevalence is almost twice as high in women compared to men (7.7 versus 4.4 per 100,000) [1]. When this occurs in postoperative patients, it presents as rapidly expanding cutaneous ulcers with violaceous, sharply demarcated edges at the sites of surgical incisions. This can lead to potentially devastating wounds and significant tissue loss leading to large areas of scarring sometimes requiring skin grafting.

According to Zuo et al., postsurgical pyoderma gangrenosum occurred most commonly in breast surgery (25%), followed by abdominal surgery (14%). The most common breast surgery was bilateral reduction mammoplasty (45%), followed by breast reconstruction (25%), as well as augmentation mammaplasty (7%) [2]. A systematic review by Erhl et al. showed that the most common associated conditions were malignancy and autoimmune disease. They also found that the microbiologic exam was negative in 90% of cases [3]. In the study by Tuffaha et al., looking at breast surgery specifically, pyoderma gangrenosum presented bilaterally in 88% of cases, abdominal involvement was seen in 6 of 7 cases where there was an abdominal donor site, and the nipples and underlying breast parenchyma were spared in 89% of wounds [4]. 

This case report demonstrates the need for a high index of suspicion for this disease process and obtaining early dermatology and infectious disease consults, as avoiding multiple debridements spared the patient from having even larger wounds and further complications.

## Case presentation

**Figure 1:**
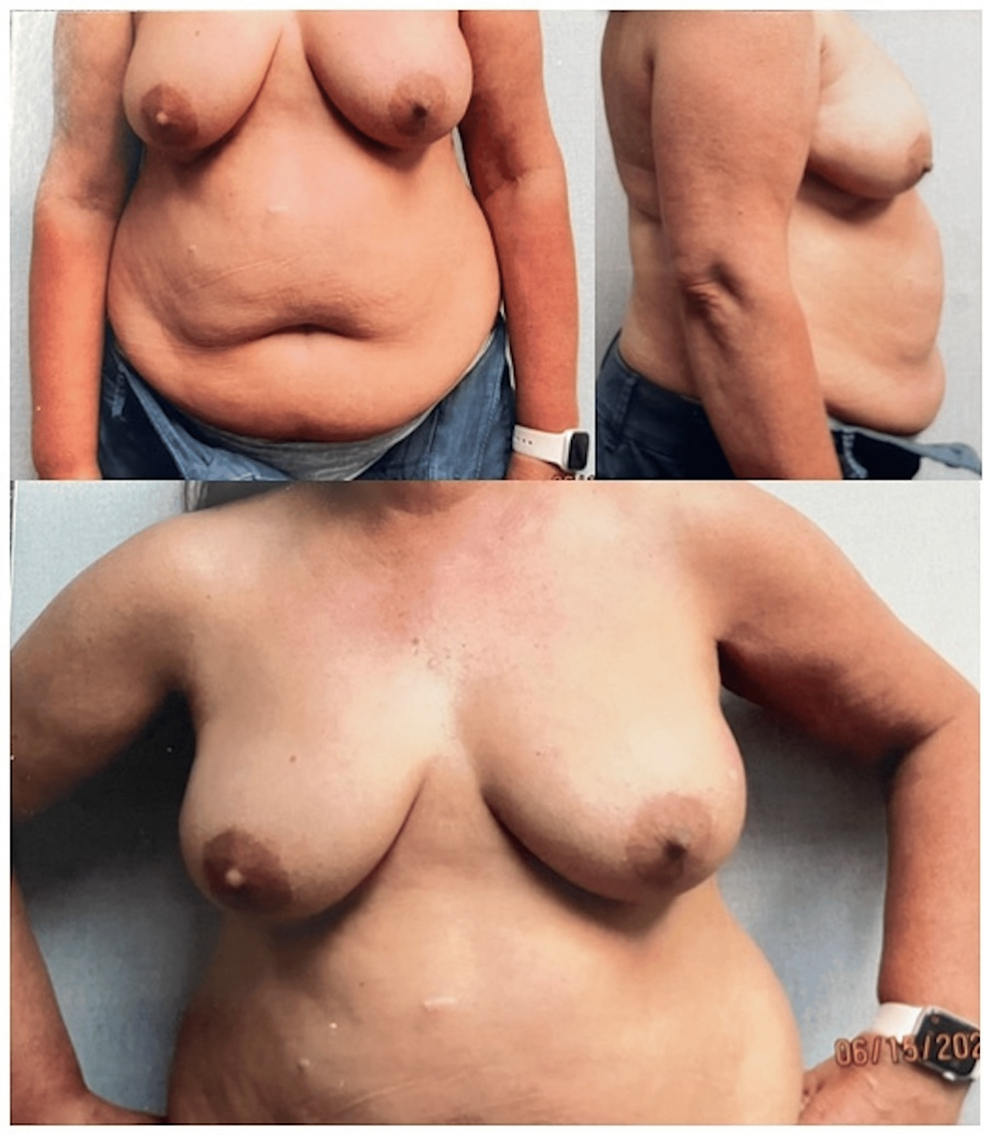
Preoperative photos.

A 55-year-old female presented in June of 2022 for consultation with bilateral breast ptosis and abdominal lipodystrophy (Figure 1).

**Figure 2:**
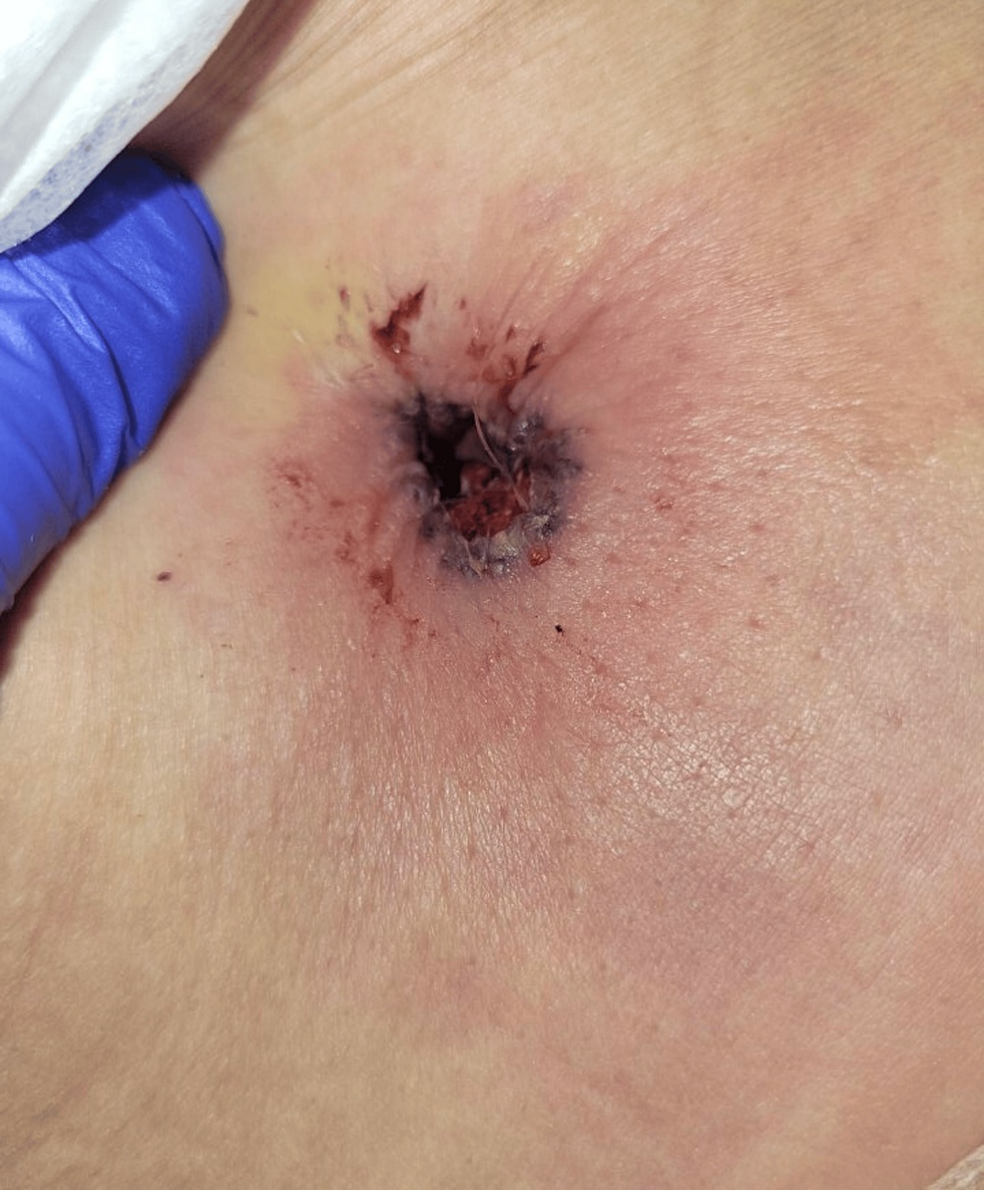
Initial presentation on postoperative day five with periumbilical pain and ecchymosis.

Her past medical history included gout and hypertension, both of which were well-controlled with allopurinol (300 mg daily) and losartan (50 mg daily). Her past surgical history included hysterectomy, tubal ligation, and needle localization breast biopsy of both breasts (pathology benign). Preoperative medical clearance, including full panel blood work and EKG, was unremarkable. She had no pertinent family history. On August 2, 2022, the patient underwent bilateral augmentation and mastopexy, abdominoplasty, and liposuction of her anterior and posterior waist without any intraoperative complications. On postoperative day five, she presented to the ED with periumbilical ecchymosis and pain (Figure 2). 

**Figure 3:**
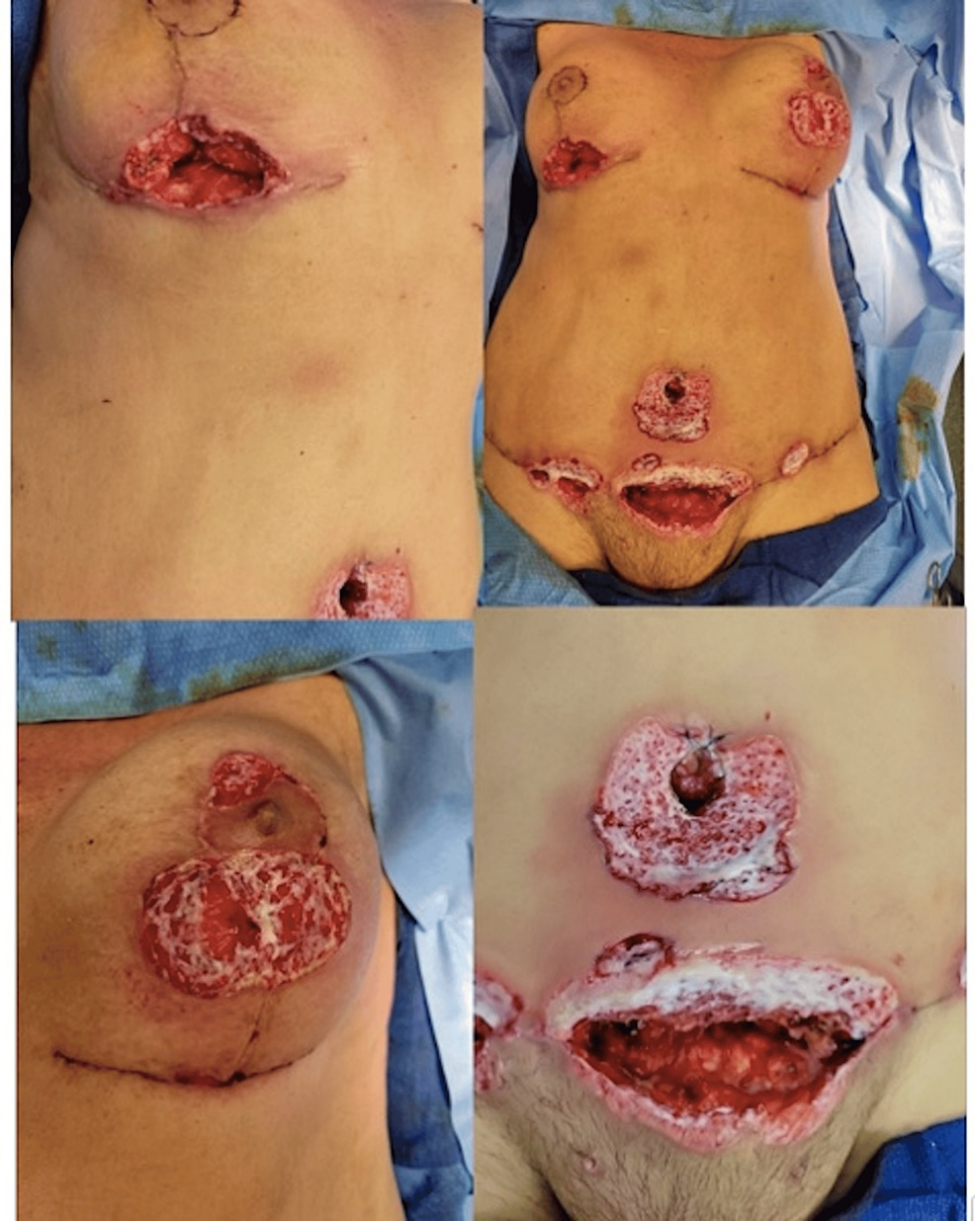
Postoperative day nine presenting with wound dehiscence abdomen, umbilicus, and bilateral breasts.

After a full workup and evaluation by the plastic surgery team along with one dose of ceftriaxone (1 g), she was discharged home on cefadroxil 500 mg BID and instructions to apply silvadene to her umbilical wound twice daily. On postoperative day nine, she presented back to the clinic with major dehiscence of abdominal, umbilical, and bilateral breast incisions (Figure 3).

Her nipple areolar complexes appeared to be mostly spared, except for a small area of the superior left nipple areolar complex. She was then admitted to the hospital with infectious disease consultation. Her lab work was significant only for a white blood cell count of 22.9 x 10^9/L and she was started on empiric IV antibiotic therapy which included vancomycin (1,000 mg every 12 hours) and piperacillin/tazobactam (3.37 g every eight hours). The following day she underwent debridement of her wounds, removal of the right breast implant, and application of negative pressure wound therapy. Multiple tissue samples and fluid were taken intraoperatively and sent for culture, sensitivity, and pathology.

**Figure 4:**
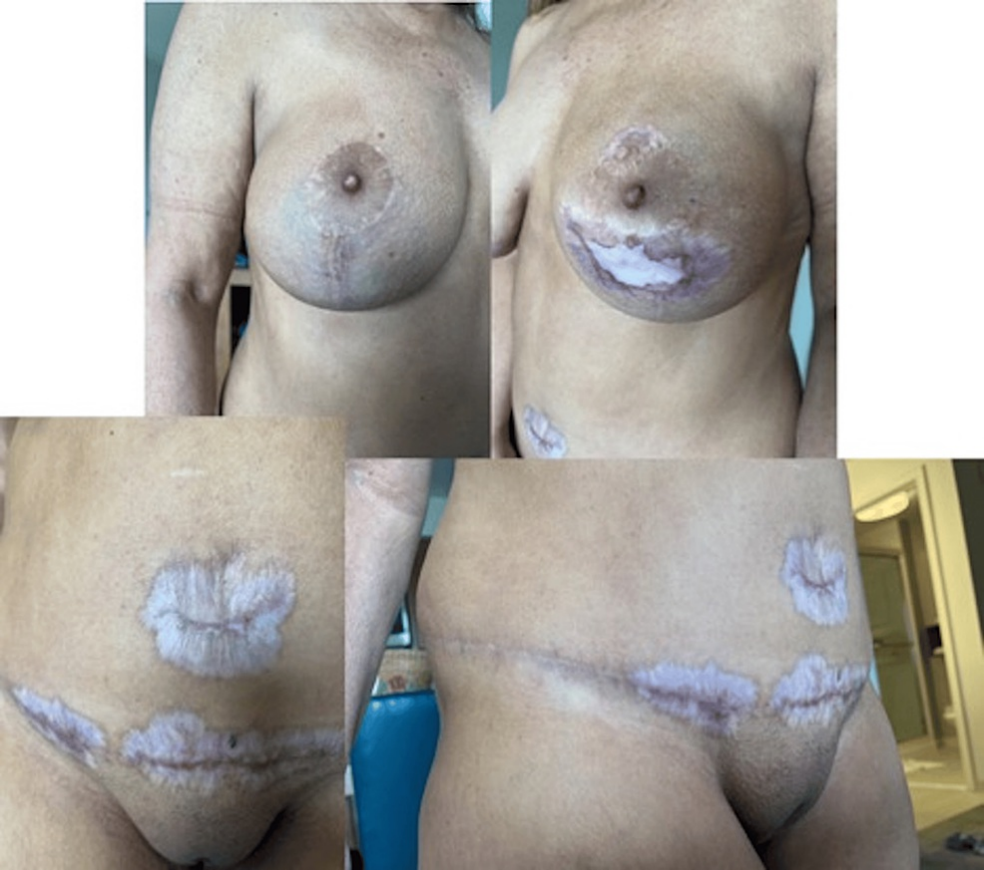
Final photos from May 2023 of all incisions healed.

**Figure 5:**
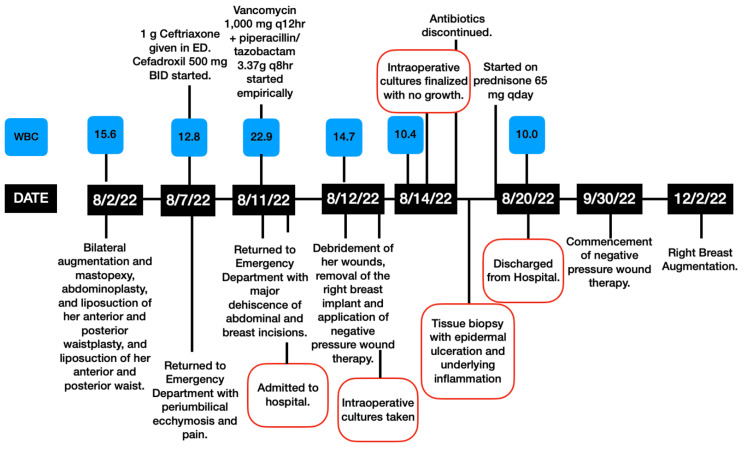
Timeline of important events, white blood cell count trend, and antibiotics administered. White Blood Cell Count listed in blue, units 10^9/L.

All of her intraoperative cultures returned with no bacterial growth and eventually, dermatology was consulted with a presumptive diagnosis of pyoderma gangrenosum. A thorough inflammatory and immunologic workup was performed. She was started on 65 mg (1mg/kg dosing) prednisone daily and instructed to apply clobetasol ointment and metronidazole gel to her wounds. Tissue biopsy resulted in epidermal ulceration with underlying dense neutrophil-rich inflammation further confirming diagnosis of pyoderma gangrenosum. Her white blood cell count normalized and her antibiotics were eventually discontinued by the infectious disease team. Dermatology recommended against further debridement and her negative pressure therapy was continued with weekly changes. She was started on cyclosporine 100 mg BID. Her wounds eventually healed after nine weeks of negative pressure therapy and local wound care. On December 2, 2022, she underwent repeat right breast augmentation She continued her immunosuppressive medications during the preoperative and perioperative period without any complications. Final results are shown in Figure 4. The timeline of critical events, white blood cell trend, and antibiotics administered are listed in Figure 5. 

## Discussion

Pyoderma gangrenosum was first described by Brocq in 1916, followed by Brunsting in 1930 [5]. Previously, diagnostic criteria have been proposed. Major criteria include a rapid progression of a painful, necrolytic, cutaneous ulcer with irregular, violaceous, and undermined borders, as well as the exclusion of other causes of cutaneous ulceration. Minor criteria include the presence of pathergy, cribriform scarring, systemic diseases associated with pyoderma gangrenosum, and histopathologic findings of a sterile dermal neutrophilia with or without mixed inflammation or lymphocytic vasculitis with necrosis or hemorrhage. A further minor criterion includes a rapid response to systemic glucocorticoid treatment, with a 50% decrease in the ulcer size at one month [6].

There are multiple subtypes of pyoderma gangrenosum noted in the literature. Various classification schemes have been described to further delineate the underlying cause including ulcerative, pustular, vegetative, bullous, peristomal, and pathergic forms of pyoderma gangrenosum. The pathergy phenomenon is defined as a state of altered tissue reactivity that occurs in response to minor trauma [7]. Termed pathergic pyoderma gangrenosum, this usually develops at a site of previous trauma, including a surgical site [8].

Postsurgical pyoderma gangrenosum has less association with systemic disease than its nonsurgical counterpart. Often, antibiotic drug therapy is erroneously initiated. Due to early misdiagnosis, the affected sites are often debrided surgically, which can lead to the progression of disease [9]. Typically when patients present, bacterial wound infection cannot be ruled out, which often leads to additional surgical procedures that are unwarranted [10]. A thorough history and physical exam should be obtained from these patients. Workup should include typical labs as well as wound cultures. 

Pathology of wound biopsies will show a central zone of necrotizing suppurative inflammation and a peripheral vascular reaction defined by perivascular and intramural lymphocytic infiltrates with a peripheral neutrophilic component, usually without concomitant fibrinoid necrosis [11].

Treatment should be initiated promptly upon diagnosis and include multimodal pain medication as well as immunosuppressive therapy. Initially, this will include systemic steroids and can be transitioned to other immunologic drugs such as cyclosporine therapy. The mode of action of cyclosporine is through binding to the enzyme cyclophilin, which causes impaired protein folding intrinsic to antigen activation of helper T lymphocytes. In doses of less than 6 mg/kg, cyclosporine takes 1-3 weeks to produce a clinical response and several months to achieve clinical remission [11].

Unnecessary debridement should be avoided; however, some debridement may be necessary to get rid of any necrotic tissue at the wound bed or borders to promote healing [12]. There are reports of patients who underwent multiple surgical debridements with the advancement of their disease, eventually requiring skin grafting due to wound size [6,12]. 

This patient continued her cyclosporine treatment while undergoing her final surgery without any recurrence of her disease or incisional breakdown following her procedure.

## Conclusions

Overall, postsurgical pyoderma gangrenosum is a rare disease that often has no association with autoimmune disorders. Often these patients are initially misdiagnosed, so a high index of suspicion is required to avoid unnecessary debridements and to promptly initiate appropriate treatment. This disease can have devastating consequences of skin and soft tissue loss if not recognized and treated in a timely fashion. 
